# Challenges in Estimating Characteristics of *Staphylococcus aureus* Nasal Carriage Among Humans Enrolled in Surveillance Studies

**DOI:** 10.3389/fpubh.2018.00163

**Published:** 2018-06-01

**Authors:** Thanh-Thao Le, Maya Nadimpalli, Jianyong Wu, Christopher D. Heaney, Jill R. Stewart

**Affiliations:** ^1^Department of Environmental Sciences and Engineering, Gillings School of Global Public Health, University of North Carolina, Chapel Hill, NC, United States; ^2^Department of Environmental Health and Engineering, Bloomberg School of Public Health, Johns Hopkins University, Baltimore, MD, United States; ^3^Department of Epidemiology, Bloomberg School of Public Health, Johns Hopkins University, Baltimore, MD, United States; ^4^Department of International Health, Bloomberg School of Public Health, Johns Hopkins University, Baltimore, MD, United States

**Keywords:** *Staphylococcus*, MRSA, antibiotic resistance, opportunistic pathogen, nasal carriage, surveillance, ROC curve

## Abstract

Evaluating carriage of *Staphylococcus aureus*, an opportunistic pathogen of humans and animals capable of causing antibiotic-resistant infections, is epidemiologically important. However, clinical and epidemiological surveillance studies of *S. aureus* typically rely on characterizing one isolate per individual, which may not represent the actual population diversity in a carrier. The objective of this study was to determine if one isolate is sufficient for determining carrier status of particular strains or characteristics of *S. aureus* in a healthy (non-hospitalized) human population. We compared *spa* types, genetic markers (*mec*A, *scn*), and antibiotic resistance profiles of 10 *S. aureus* isolates recovered from a single nasal swab for each of 19 participants (190 isolates total) selected from a cohort of industrial hog operation workers and their household members. Participants included both persistent (*n* = 10) and intermediate (*n* = 9) carriers of *S. aureus*. Among the participants, 17 (89%) carried a single *S. aureus spa* type intranasally and the other two carried dominant *spa* types. Less similarity was observed for genes encoded on mobile genetic elements (*mec*A, *scn*) and antibiotic resistance profiles. Statistical modeling, based on receiving operating characteristic curves, suggests that three to five isolates may be necessary to accurately assign nasal carriage status for these more variable characteristics. Variability was observed for both persistent and intermediate carriers of *S. aureus*. These results suggest that surveillance studies that rely on testing one *S. aureus* isolate are likely to identify predominant *spa* types but may not fully capture more variable characteristics of *S. aureus*, including antibiotic resistance. Surveillance studies that rely on testing one isolate may underestimate prevalence of nasal carriage of *S. aureus* with these more variable characteristics.

## Introduction

*Staphylococcus aureus* is an opportunistic pathogen that can colonize the nasal cavities of humans and animals and can cause superficial or life-threatening infections. Infections caused by *S. aureus* include skin and soft tissue infections, bacteremia, endocarditis, gastrointestinal illness, necrotizing pneumonia, post-operative infections, and toxic shock syndrome (1, 2). In the United States, annual incidence of *S. aureus* bacteremia is 4.3–38.2 per 100,000 person years; and the 30 day all-cause mortality rate is approximately 20% (3).

Infections caused by antibiotic-resistant strains of *S. aureus* including methicillin-resistant *S. aureus* (MRSA) and multidrug-resistant *S. aureus* (MDRSA) are particularly troublesome as they can be more difficult and costly to treat (4). These infections have reached epidemic proportions globally (5), and are a leading concern in the global fight against antibiotic resistance (6). MRSA first emerged in hospital settings in the 1960s and predominantly affected elderly and sick patients (2). However, MRSA is no longer confined to the hospital setting. Antibiotic resistant *S. aureus* strains including MRSA are increasingly detected in the community (7, 8), and among healthy individuals with livestock contact including livestock operation workers and veterinarians (9–14). Systematic surveillance is needed within and outside of hospitals and following a “One Health” approach (15) to better understand the ecology of emerging strains.

Most surveillance studies rely on characterizing a single *S. aureus* isolate from a nasal swab per individual (16). Exceptions include studies that test more than one anatomical site per participant or longitudinal studies that test participants over time. However, even these studies typically rely on testing a single *S. aureus* isolate per sample. It is not clear whether testing one isolate per sample is sufficient for surveillance of particular characteristics of *S. aureus*, including tests for multidrug resistance. A recent study of MRSA transmitted among staff and animal patients at a veterinary hospital demonstrated considerable within-host genetic diversity of MRSA during carriage and infection (17), and co-colonization of humans by distinct *S. aureus spa* types has been reported (18, 19). Surveillance studies are often reviewed for their sampling procedures including the representativeness of participating populations. However, less attention is paid to procedures applied to test for carriage in individual participants. Also, less attention tends to be paid to healthy populations though healthy individuals can act as carriers for antibiotic resistant bacteria, facilitating their spread and diversification.

The objective of this study was to determine whether analysis of a single *S. aureus* isolate from an individual's anterior nares is adequate to determine carrier status of a particular *spa* type or *S. aureus* characteristic. We characterized 10 rather than one *S. aureus* isolate for each participant and evaluated all 10 isolates for characteristics commonly tested in surveillance studies. Characteristics tested in this study include occurrence of *mec*A, a gene that confers resistance to β-lactam antibiotics including methicillin that is often used to indicate MRSA carriage; and occurrence of *scn*, a gene that serves in evasion of the human innate immune response and whose absence is sometimes used to indicate livestock association (20). All isolates were also tested for resistance to a panel of 15 antibiotics used in human and/or animal medicine (11). Among characteristics for which clonality was not observed, our secondary objective was to use mathematical models to determine the number of isolates needed to correctly characterize an individual's *S. aureus* nasal carrier status.

## Materials and methods

Study participants (*n* = 19) were selected from a cohort of 103 industrial hog operation (IHO) workers and 80 household members who participated in a repeated-measures study to assess persistence of *S. aureus* nasal carriage (11). The JHSPH Institutional Review Board (IRB) approved this study (IRB00004608) and the UNC Non-Biomedical IRB approved reliance on the JHSPH IRB. In the cohort study, nasal swabs were obtained from each participant's anterior nares using a BD BBL™ CultureSwab™ once at baseline then every 2 weeks during a 4-month follow-up period. Individuals were classified as persistent carriers, intermediate carriers, or non-carriers of *S. aureus*. Persistent carriers had at least 80% of their nasal swabs test positive for *S. aureus* while intermittent carriers had <80% but more than 0% test positive for *S. aureus*. Non-carriers tested negative for *S. aureus* nasal carriage throughout the study.

For this sub-study, one nasal swab from each of 10 persistent and nine intermittent carriers, including both IHO workers (*n* = 11) and members of their households (*n* = 8; Table 1), was screened to characterize multiple isolates of *S. aureus* from the same source. The swabs were collected between a participant's third and eighth biweekly study visits. Ten *S. aureus* isolates were characterized from each of the 19 participants, and thus a total of 190 isolates were tested.

**Table 1:** *Spa* types of *S. aureus* (*n* = 10 isolates) recovered from nasal swabs collected from each of 19 participants.

	**Participant no**.	**IHO worker/Household member**	***spa* types detected (# of isolates)**
Persistent carriers	1	IHO worker	t3446 (10)
	2	IHO worker	t034 (10)
	3	IHO worker	t337 (10)
	4	IHO worker	t002 (10)
	5	IHO worker	t337 (10)
	6	Household member	t5739 (10)
	7	Household member	t5739 (10)
	8	Household member	t034 (10)
	9	Household member	t089 (10)
	10*	Household member	t7226 (8), t002 (2)
Intermittent carriers	11*	IHO worker	t337 (9), t14243 (1)
	12	IHO worker	t14157 (10)
	13	IHO worker	t233 (10)
	14	IHO worker	t5883 (10)
	15	IHO worker	t645 (10)
	16	IHO worker	t337 (10)
	17	Household member	t233 (10)
	18	Household member	t1937 (10)
	19	Household member	t3802 (10)

IHO, industrial hog operation. Participants 10 and 11, marked with “*,” carried two different spa types.

To isolate *S. aureus* colonies, each nasal swab was clipped into a micro-centrifuge tube containing 1 mL of phosphate-buffered saline and vortexed for 1 min. From this suspension, 100 μL was spread plated onto CHROMagar™ *S. aureus* and incubated at 37°C for 18–24 h. Colonies with morphological characteristics of *S. aureus* were counted and 10 colonies that appeared easiest to isolate without touching other colonies were selected, streaked to isolation, and analyzed for genetic and antibiotic resistance characteristics.

Multiplex polymerase chain reaction (PCR) was conducted to identify the staphylococcal protein A gene (*spa), mec*A, and *scn* (11, 21). *Spa* is a marker for *S. aureus*, and the presence of both *spa* and *mec*A is indicative of MRSA. Absence of the human immune evasion cluster *scn* gene is an indicator of livestock association (11, 20). For each isolate, *spa* typing was performed as described elsewhere (22) using the Ridom Staph Type software and the Ridom SpaServer (http://spa.ridom.de/index.shtml).

**Figure 1:**
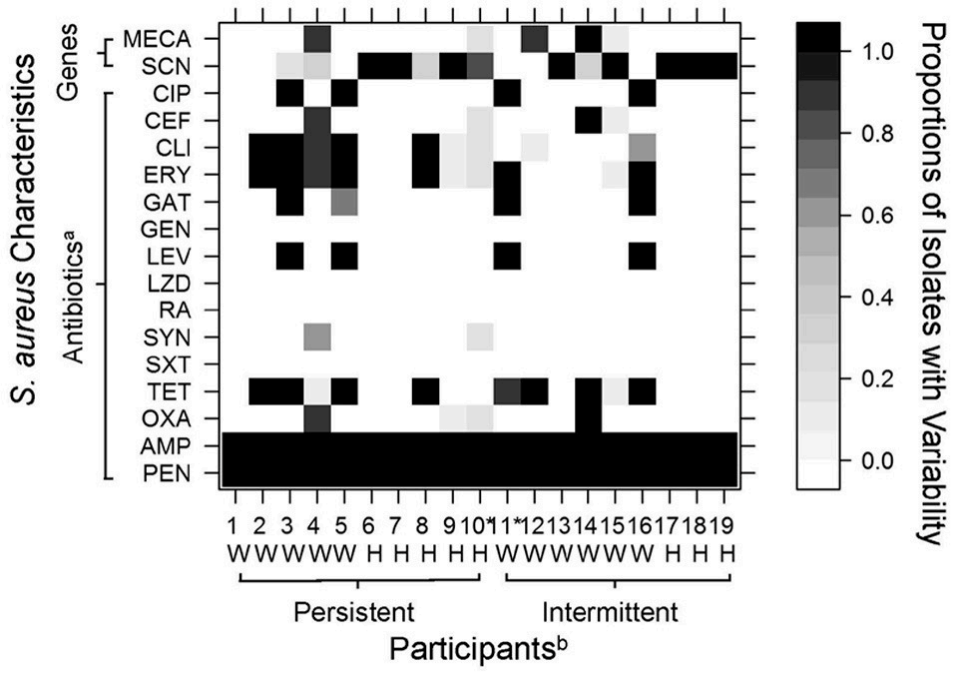
Variability among 10 *S. aureus* isolates recovered from nasal swabs collected from 19 industrial hog operation (IHO) workers and their household members. Variation in color indicates the observed diversity in tested characteristics, with white cells denoting that no isolates were resistant to a tested antibiotic or positive for a particular gene (i.e., *mec*A, *scn*), black cells denoting that all 10 isolates were resistant or positive for a particular gene (i.e., *mec*A, *scn*), and gray cells denoting variability among isolates as shown in the legend. ^a^CIP, ciprofloxacin; CEF, ceftriaxone; CLI, clindamycin; ERY, erythromycin; GAT, gatifloxacin; GEN, gentamicin; LEV, levofloxacin; LZD, linezolid; RA, rifampin; SYN, quinupristin/dalfopristin; SXT, sulfamethoxazole with trimethoprim; TET, tetracycline; OXA, oxacillin; AMP, ampicillin; PEN, penicillin. *mec*A, methicillin resistance gene; *scn, scn* human immune evasion cluster gene. ^b^W, Industrial Hog Operation (IHO) Worker; H, Household Member. *Participants 10 and 11 carried two different *spa* types. Other participants each carried a single *spa* type.

The Kirby-Bauer disk diffusion method was used to assess each isolate's susceptibility to a panel of 15 antibiotics (Figure 1). Interpretations were based on Clinical and Laboratory Standards Institute (CLSI) guidelines (23). Induced clindamycin resistance was determined by the presence of a D-zone with erythromycin resistance (24). For this study, we defined isolates that demonstrated intermediate to complete resistance to an antibiotic as “resistant.”

Receiver operating characteristic (ROC) curves were generated to compare the performance of different sampling scenarios needed to adequately characterize *S. aureus* carried intranasally. This analysis is commonly considered an effective approach for assessing the performance of diagnostic tests (here, sampling scenarios). The area under the ROC curve (AUC) is an index for measuring performance, with a larger AUC indicating a better performance (e.g., more accurate to determine the carrier status). If at least one of the tested isolates from an individual was positive for a particular gene or resistant to a particular antibiotic, we considered that person to be a true carrier of that specific characteristic. We generated three scenarios utilizing information from one, three, and five colonies, using the results from testing 10 colonies as a reference, and calculated the AUC for each sampling scenario. We considered an AUC <50% to correspond to random chance that an individual would be identified as a true carrier and an AUC >90% to indicate that the number of isolates tested was sufficiently accurate to determine the carrier status (25). ROC curves were drawn and AUCs were calculated using the “pROC” package (26) in the R statistical package, Version 3.2.1 (The R Foundation, Vienna, Austria). We were not able to generate ROC curves for the *spa* types because the data are not binary. Instead, we calculated positive probabilities to estimate whether testing a single isolate would have identified the dominant *spa* type.

## Results

Ten *S. aureus* isolates were characterized from each of 19 participants (190 isolates total) including both persistent and intermediate carriers. For 17 of 19 (89%) participants, *spa* types of all 10 isolates were identical (Table 1). The remaining two participants carried two distinct *spa* types; but both had a predominant *spa* type and probability analysis suggests that testing one isolate would have been adequate in most cases. If we had tested one colony among our participants, the positive probability for identifying the dominant strain would have been 100% for 17 participants, 90% for one participant, and 80% for one participant, leading to an overall accuracy of 98%.

Variability in genes encoded on mobile genetic elements (*mec*A, *scn*) or antibiotic resistance profiles was observed among 11 (58%) of the 19 study participants (Figure 1). Four participants (21%) carried *S. aureus* that diverged in *mec*A presence. These four participants included two persistent carriers (one worker and one household member) and two intermittent carriers (one worker and one household member). Both workers had nine isolates that were positive for *mec*A and one isolate each that was negative. Both household members had one or two isolates positive for *mec*A and the remaining testing negative.

Five participants (26%) carried *S. aureus* that demonstrated variability in absence of *scn*, commonly used as a marker for livestock-association (Figure 1). These participants included four persistent carriers (two workers and two household members) and one intermittent carrier (a worker). Additionally, eight participants (42%) demonstrated variability in antibiotic resistance patterns (Figure 1). Variability of resistance patterns of intranasal *S. aureus* was again observed among both persistent and intermittent carriers (four participants each among persistent and intermittent carriers), and among both IHO workers and household members. Six workers and two household members showed variability in resistance patterns.

Using ROC curves, we were able to estimate the number of isolates needed to accurately determine carrier status for particular genes encoded on mobile genetic elements (*mec*A, *scn*) or phenotypic antibiotic resistance. Results suggest that testing five isolates from an individual's nasal swab would have been adequate to determine carriage of *mec*A, often used to define MRSA nasal carriage (Table 2). Three isolates would have been adequate to determine carriage of *scn*. Depending on the antibiotic assessed, different thresholds would have been needed to determine resistance (Table 2). For example, testing five isolates appeared necessary to determine clindamycin resistance, while one isolate would have been adequate to determine resistance to tetracycline, ciprofloxacin or levofloxacin. Individuals who carried isolates that diverged in resistance patterns tended to show divergence across multiple antibiotics. Participants 4, 9, 10, and 15 in particular carried isolates with variable and divergent antibiotic resistance patterns as demonstrated by the gray cells in Figure 1.

**Table 2:** Area under the curve (AUC) values for Receiver Operating Characteristic (ROC) curves used to assess number of isolates needed to determine nasal carriage of *S. aureus* with particular characteristics.

	***S. aureus* characteristics**	**Area under the curve (%)**		
		**No. of isolates tested in sampling scenario**		
		**1**	**3**	**5**
Genes	*mec*A	81.4	87.8	91.2^*^
	*scn*	88.0	93.4^*^	96.4^*^
Antibiotic^b^	CIP	100^*^	100^*^	100^*^
	CEF	80.5	91.5^*^	95.8^*^
	CLI	83.0	87.5	91.2^*^
	ERY	86.0	89.5	92.3^*^
	GAT	72.3	78.2	85.3
	GEN^a^	–	–	–
	LEV	100^*^	100^*^	100^*^
	LZD^a^	–	–	–
	RA^a^	–	–	–
	SYN	68.0	86.2	90.0^*^
	SXT^a^	–	–	–
	TET	90.3^*^	92.4^*^	93.7^*^
	OXA	69.8	81.2	84.2
	AMP^a^	–	–	–
	PEN^a^	–	–	–

An AUC >90% (*) indicated that the number of isolates tested was sufficient to capture the population diversity (spa types, genotypes, and drug resistance patterns) of S. aureus in tested nasal samples.

a *Antibiotics with a “–” denote that all of the collected isolates from each participant were the same so an AUC value could not be calculated*.

b *Intermediate or complete resistance to: CIP, ciprofloxacin; CEF, ceftriaxone; CLI, clindamycin; ERY, erythromycin; GAT, gatifloxacin; GEN, gentamicin; LEV, levofloxacin; LZD, linezolid; RA, rifampin; SYN, quinupristin/dalfopristin; SXT, sulfamethoxazole with trimethoprim; TET, tetracycline; OXA, oxacillin; AMP, ampicillin; PEN, penicillin*.

## Discussion

In this study we demonstrated that genetic characteristics and antibiotic resistance patterns can vary among *S. aureus* isolated from a single nasal swab, although genotypes including *spa* types of *S. aureus* appear to be relatively stable per individual at a single point in time. Our results are consistent with previous studies that characterize *S. aureus* from human nares. In our study, two of 19 individuals (10.5%) carried two rather than one *spa* type of *S. aureus*. An earlier study of healthy individuals reported that up to 5.8% of 360 participants were co-colonized with different *S. aureus spa* types at any given time and that 18% were co-colonized at least once over a 2 year study period (19). Results appear similar even when other genotyping methods are employed. A study using pulsed field gel electrophoresis (PFGE) to evaluate strain diversity estimated that ~6.6% of colonized individuals would be expected to carry multiple genetic strains (18). Nasal colonization with different MRSA subtypes has also been observed, both in hospitalized human patients (27, 28), and in humans and animal patients during an outbreak that occurred as a veterinary hospital (17). This latter study concluded that multiple isolates from individuals need to be analyzed to better understand disease ecology and transmission networks. Our study further suggests that analysis of multiple isolates may be advisable to evaluate carrier status of particular characteristics of *S. aureus*.

Our study is the first to our knowledge to apply statistical modeling to determine the number of isolates required to determine the carrier status for particular *S. aureus* characteristics (i.e., genotypes, subtypes, or resistance profiles). This approach was effective in capturing the variation in *S. aureus* characteristics despite the limitation of a small sample size. Further, nesting of this study within a larger cohort study enabled us to choose participants based on prior knowledge of their carrier status. We were able to document that both persistent and intermediate carriers of *S. aureus* sometimes carry strains with different genetic characteristics or drug resistance patterns. These results can have implications from both a clinical perspective (e.g., deciding appropriate antibiotic treatment) and an epidemiological perspective (e.g., evaluating prevalence of particular strains in populations). Differences between individuals in this study were also notable, with some participants showing no variation in characteristics among tested isolates while other participants showed variation in multiple characteristics (gene markers or antibiotic resistance). Although it was beyond the scope of this study, it would be interesting to test whether these variations could be associated with host characteristics, environmental exposures, or other factors.

Our results suggest that the number of isolates necessary for surveillance studies depends on study objectives including what *S. aureus* characteristic is most relevant epidemiologically and/or clinically. The answer may vary depending on whether the goal is to determine *spa* type, MRSA carriage, livestock association, or clinical treatment based on antibiotic resistance profiles. Characterization of one isolate appears sufficient to accurately capture relatively stable characteristics of *S. aureus* among nasal carriers, particularly to identify the *spa* type of *S. aureus* among nasal carriage positive individuals. However, testing of additional isolates appears necessary for more plastic characteristics including the occurrence of genes encoded on mobile genetic elements (*mec*A, *scn*) routinely used to determine MRSA carriage and livestock association, respectively, and phenotypic antibiotic resistance profiles. Depending on the characteristic of interest, our analysis suggests that one to five isolates may need to be tested to accurately evaluate nasal carriage of *S. aureus* with these more plastic characteristics. It is concluded that by assuming *S. aureus* colonization by a single strain in the nares, surveillance studies that rely on testing one isolate may underestimate nasal carriage of these more variable *S. aureus* characteristics.

## Ethics statement

The Johns Hopkins School of Public Health (JHSPH) Institutional Review Board approved this study (IRB00004608) and the University of North Carolina Non-Biomedical IRB approved reliance on the JHSPH IRB. Before participating, adults provided written informed consent. Written parental permission and informed assent were collected for participants seven to 17 years of age.

## Author contributions

CH, JS, and MN contributed to the conceptualization and design of this study, and JS and CH were responsible for funding acquisition and project administration. T-TL and MN were actively involved in data collection, with JW joining them for data curation and analysis. All authors contributed to the writing and editing of this manuscript, and approved final submission.

### Conflict of interest statement

The authors declare that the research was conducted in the absence of any commercial or financial relationships that could be construed as a potential conflict of interest.
